# Conflict or Collaboration—The Impact of Knowledge Endowment Heterogeneity on Remix in Open Collaborative Communities

**DOI:** 10.3389/fpsyg.2022.941448

**Published:** 2022-06-16

**Authors:** Juan Tan, Congcong Qi, Xiaohui Gao, Jianle Lu, Qiong Tan

**Affiliations:** ^1^Business School, Beijing Technology and Business University, Beijing, China; ^2^School of Economics, Central South University of Forestry and Technology, Changsha, China

**Keywords:** conflict, collaboration, knowledge endowment, open collaborative community, remix

## Abstract

With the emergence of online open platforms and communities, remix has drawn much attention as an essential source of innovation whereby the knowledge endowment of online community users plays a crucial role. This study constructs a structural equation model to explore the impact of user knowledge endowment heterogeneity on remix through the mediating effect of their collaborative psychology. In this empirical study, we collected 25,032 pieces of data from Thingiverse (a 3D printing community) users and their published designs. The findings are as follows. Explicit knowledge endowment heterogeneity has a positive impact on the quantity of remix but a negative impact on its quality. Likewise, the implicit knowledge endowment heterogeneity positively affects the quantity of remix but has no significant effect on its quality. Users’ conflicting psychology plays a mediating role between knowledge endowment heterogeneity and remix, while their collaborative psychology negatively mediates merely between explicit knowledge endowment heterogeneity and remix quality. By unraveling the relationship between user knowledge endowment heterogeneity, collaborative psychology, and remix, this study is significant in understanding users’ remix process in open collaborative communities and illuminating their psychological mechanism in this process.

## Introduction

The source of innovation remains a focus in management studies in recent years (Rosenberg, 1982; Von Hippel, 1988; Van de Ven et al., 1999; Hargadon, 2003; Simonton, 2004; Usher, 2011). It is generally believed that innovation does not take place in isolation but involves the recombination of existing “elements” more or less (Schumpeter, 1942; Nelson and Winter, 1982; Van de Ven, 1986; Weitzman, 1998; Arthur, 2009; Salter and Alexy, 2014). In particular, remix has gained more and more attention as online open platforms and communities come into being (Lessig, 2008; Khatib et al., 2011; Tuite and Smith, 2012; Cheliotis et al., 2014; Sapsed and Tschang, 2014; Oehlberg et al., 2015; Dasgupta et al., 2016; Stanko, 2016). It refers to the rearrangement and reuse of existing elements. Remixes are defined as “works created through changing and combining existing works to produce something new and creative” in the U.S. Department of Commerce’s Green Paper on Copyright Policy, Creativity, and Innovation in the Digital Economy dated 2013. Terms such as remix, recombination, and reuse are often used interchangeably in related studies. Open Collaboration Community (OCC) offers a vital foundation for remixing whereby innovators can apply existing ideas to new environments, recombine them or incorporate some of them into their own innovation. OCC users can not only “passively consume” the innovations of others but also have the right to innovate by remixing the works of others (Cheliotis, 2009; Flath et al., 2017). According to Faraj et al. (2011), OCC is a space for instant collaborative innovation, and the development thereof is propelled by users’ sharing of their own knowledge with others or integrating others’ knowledge, offering a variety of solutions to specific innovation difficulties, and working collaboratively toward innovation. Nonetheless, some studies reveal that OCC users are hardly enthusiastic about innovation and need to improve the quality of remixes that they publish (Stanko, 2016; Flath et al., 2017). It is, therefore, vital to stimulate OCC users’ potential and encourage them to engage in knowledge innovation constructively in order to maintain the sustainable development of OCC.

Existing studies on the impact of OCC knowledge management on remix patterns are mainly dedicated to the classification of user-generated content (Flath et al., 2017), which attempt to explore the motivation, willingness, and behavior of users in knowledge sharing and innovation (Hill and Monroy-Hernández, 2013; Stanko, 2016). Little attention has been paid to the impact of user knowledge endowment on remix in two aspects. Firstly, while some scholars analyze the attributes of user-generated content (Dasgupta et al., 2016; Stanko, 2016) and the impact of user-generated content heterogeneity on remix performance (Kim et al., 2016; Flath et al., 2017; Voigt, 2018), they fail to probe in the mechanism of user knowledge endowment on remix. Secondly, the relationship between the diffusion (Stanko, 2016; Tan et al., 2020), transfer (Flath et al., 2017), and spillover (Kyriakou et al., 2017) of knowledge in OCC and user remix can account for the advantages of user remix derived from the heterogeneous knowledge endowment, albeit the process mechanism of user knowledge endowment in driving remix remains unclear.

Studies have established that the knowledge endowment heterogeneity between users can provide rich innovation resources to OCC. Meanwhile, users’ proactive participation in “knowledge development,” knowledge exchange and innovation, and collaborative interaction in knowledge enables reciprocal sharing of knowledge between different OCC users and groups and thus amplifies synergies (Yang and Hou, 2020). Therefore, based on explicit and implicit knowledge endowment heterogeneity, the present study explores the mediating role of users’ collaborative psychology between knowledge endowment heterogeneity and remix, reveals the acting path of users’ collaborative and conflicting psychology, respectively, and finally builds a structural equation model for investigating the effect of OCC users’ knowledge endowment heterogeneity on remix. This study is expected to enrich the study on the knowledge activities preceding remix in theory and offer recommendations for stimulating the enthusiasm of OCC users in practice.

## Literature Review and Research Hypothesis

### Knowledge Endowment Heterogeneity and Remix

Knowledge endowment heterogeneity refers to the effective use of heterogeneous knowledge endowment between community users to remove the constraints in innovation rules and explore the key resources vital for new opportunities for product development under the constraint of existing inertial thinking (Amabile et al., 1996). According to Ikujiro Non-aka’s classification criteria for corporate knowledge, we classify knowledge endowment heterogeneity into explicit and implicit ones (Polanyi, 1958). Specifically, the former refers to the differences in expertise, methods, and other aspects, while the latter indicates the differences in work experience, logical thinking, etc. between users (Yan, 2014).

OCC users are encouraged to remix the knowledge products of other users, instead of remixing their own. The explicit knowledge endowment displayed by OCC users amid remix can enrich the resources of remix, help users to discover and match the source knowledge of “remixes” more effectively, and prepare for more remixes by evaluating and developing the source knowledge (Majchrzak et al., 2004). Therefore, we propose the following hypotheses:

H1a: Explicit knowledge endowment heterogeneity has a positive impact on the quantity of remix.

According to the social categorization theory, community users with the larger explicit knowledge endowment heterogeneity tend to categorize consciously or unconsciously, and prefer the users similar to themselves, thereby forming a hierarchy and different groups within the community (Horwitz and Horwitz, 2007; Aparna and Hyuntak, 2009). This further leads to identity within groups and bias against members outside the groups. Specifically, Thingiverse users prefer remixing the works of other members within the groups, thus hindering their communication with other groups of users and resulting in the formation of “information cocoons.” However, one of the core values of remix lies in addressing the defects of existing innovations and optimizing innovations for specific applications (Kim et al., 2016). As such, the communication barrier between user groups is unfavorable to the rapid iterative innovation in knowledge products and is likely to result in a decline in remix quality. Therefore, we propose the following hypotheses:

H1b: Explicit knowledge endowment heterogeneity has a negative impact on the quality of remix.

The implicit knowledge endowment *per se* is hard to identify. In this study, we measure it by looking at the role of users within the community and groups they join. In fact, various products of OCC users involve multiple areas of knowledge. Generally speaking, users are reluctant to create new products in the knowledge domains which they are not familiar with (Friesike et al., 2019). Yet, for the purpose of remix, users will instinctively break their autognosis and search for knowledge elements that can be used for remix throughout the community. In this way, users can step into many “blind areas” and “unknown areas” of ontological knowledge. Meanwhile, as users play more roles and join more groups in this process, they will have a broader horizon and generate more knowledge links (Ransbotham et al., 2012; Foss et al., 2020). All these create favorable conditions for remix. In addition, in terms of knowledge flows, as community users play more roles and join more groups, it is more conducive to the flow of community knowledge between different areas, which will further create favorable conditions for remix based on diverse knowledge and enable users to create more premium remixes. Therefore, we propose the following hypotheses:

H2a–H2b: The implicit knowledge endowment heterogeneity has a positive impact on remix (both in quantity and quality).

### User Knowledge Endowment Heterogeneity and Collaborative Psychology

Knowledge collaboration is a process of ultimately generating knowledge innovations after the interaction between knowledge subjects and knowledge objects in time and space, as well as knowledge flow (Tong et al., 2019). In the knowledge management characterized by “knowledge collaboration,” the synergy and interaction of knowledge are fulfilled through practice, learning, interest, and target communities for the purpose of collaboration, sharing, and cooperative innovation. Knowledge collaboration is a psychological mechanism whereby participants strive to mine and utilize internal and external resources for knowledge creation and finally produce valuable achievements. Hierarchically, it can be classified into individual, group, and organizational collaborative psychology (Tong et al., 2019). The present study addresses the collaborative psychology of individual users specifically. Ikujiro Nonaka is well-known for his study on the site of knowledge innovation, namely Ba. In particular, he argues that each site provides a base that facilitates knowledge transformation and accelerates knowledge innovation (Polanyi, 1958). As an important base of knowledge innovation in the Internet era, OCC covers the Originating Ba, Interacting Ba, Cyber Ba, and Exercising Ba. Within the base, OCC users can maintain frequent information exchange and knowledge interaction through various means, thus giving full play to the efficacy of the psychological mechanism of knowledge collaboration. The collaborative and conflicting psychology of users plays an important role, respectively. The collaborative psychology of OCC users in remix refers to a process of knowledge creation and knowledge network formation by OCC users willing and motivated to work together through interaction, interplay, and coordination (Ensley and Pearce, 2001). The knowledge endowment heterogeneity is an essential source of diverse knowledge resources. Core and peripheral community users with different experiences and skills might have different but complementary attitudes and viewpoints on the same cognitive object (Safadi et al., 2021), which will facilitate the knowledge sharing between them (Renzl, 2008) and create atmospheres and conditions for the formation of collaborative psychology for remix. At the same time, individual cognition and group cognition will influence each other, and influenced by group cognition, individual users might change their own cognition, form group identity, or integrate individual and group cognition (Qiu and Wang, 2018). This interaction between individuals and groups also enables the collaborative psychology amid remix. Therefore, we propose the following hypotheses:

H3a–H3b: User knowledge (explicit and implicit) endowment heterogeneity has a positive impact on collaborative psychology.

The conflicting psychology in remix is embodied in the differences, collisions, and even confrontations between OCC users arising from the knowledge endowment heterogeneity (Zhang and Ni, 2007). Due to the differences in knowledge backgrounds, cognitive abilities, and so on, OCC users may have conflicting psychology during remix, which will further result in conflicts and differences in views and opinions (Zhang and Ni, 2007). According to the cognitive development theory, community users with different knowledge backgrounds and ways of thinking generally have differences in the same cognitive task. When they cannot assimilate and adapt to cognitive differences, the cognitive balance will be broken, hence the conflicting psychology (Jehn et al., 1997). Obviously, knowledge endowment heterogeneity is an important factor that gives rise to cognitive differences between users. The same product has different values for different users, which may cause value conflicts. Such conflicts are reflected in users’ different judgment of use values of remixes as well as their different willingness to allow other users to remix their works and generate the exchange value. Furthermore, as mentioned above, the knowledge endowment heterogeneity may also contribute to misexpression and misunderstanding between users, thus hindering the effective transfer of knowledge and enhancing their conflicting psychology (Nan and Li, 2019). Therefore, we propose the following hypotheses:

H4a–H4b: The user knowledge (explicit and implicit) endowment heterogeneity has a positive impact on the conflicting psychology.

### Synergistic Psychological Mechanism and Remix

According to the strong reciprocity theory (Gintis, 2000), some OCC users can make “selfless” contributions and demonstrate “community citizenship behaviors,” which will consolidate their collaborative psychology during remix. In accordance with the norm of reciprocity in social interaction, individuals tend to perform reciprocal behaviors of helping others whoever helped them. Based on this theory, we hold that community users who have ever received support and help have stronger psychology of participation in collaboration. Specifically, after acquiring valuable knowledge in remix, individual users will have stronger collaborative psychology and tend to publish and share more premium works with other community users. In addition, collaborative psychology is conducive to users’ active learning, thereby generating and disseminating derivative knowledge more vigorously during remix. Therefore, we propose the following hypotheses:

H5a–Hb: Users’ collaborative psychology has a positive impact on remix in both quantity and quality.

The conflicting psychology in remix is the basis of “creative tension,” “creative conflict,” or “creative chaos,” which are the sources and cradles of new knowledge, methods, and ideas (Amason, 1996; Zhang and Ni, 2007). Therefore, properly stimulating the conflicting psychology in remix contributes to more new forms of knowledge fusion and creation. Some existing studies show that team conflicts and divergences usually cause diversity and promote team innovation (Tjosvold and Su, 2007). It is further confirmed that task conflicts can promote innovation, but relationship conflicts may inhibit innovation. Task conflicts can promote innovation only in an open and inclusive language environment (Baron, 1984; Farh et al., 2010; Bradley et al., 2012). OCC features an open and inclusive atmosphere for innovation. If remix is considered as a teamwork, these teams are more involved in task conflicts rather than interpersonal conflicts, and to a certain extent, this will create more remixes. Therefore, we propose the following hypotheses:

H6a: Users’ conflicting psychology has a positive impact on the quantity of remix.

However, users’ conflicting psychology in remix does not always have a positive impact. Since the works published by OCC users are hard-won, they usually have a stronger sense of belonging toward such works (Kim et al., 2016). In contrast, remixes are usually generated by copying and modifying the existing works of other OCC users. Therefore, community users with a stronger sense of belonging may directly reject others’ remix requests and are more likely to have conflicting psychology within the community (Hill and Monroy-Hernández, 2013). Experiential evidence shows that the works of OCC users with a stronger sense of belonging usually feature novelty, high technical content, and high popularity. Obviously, the conflicting psychology of such users is unfavorable to an improvement in the overall remix level of community users. Moreover, according to the self-determination theory (Deci and Ryan, 2008), community users are more willing to self-learning and self-improvement only when they are supported by the community. However, the conflicting psychology, to a certain extent, will hinder the communication between users, reduce their satisfaction with community support, and diminish their pursuit of premium remixes. Therefore, we propose the following hypotheses:

H6b: Users’ conflicting psychology has a negative impact on the quality of remix.

### The Mediating Effect of Collaborative and Conflicting Psychology

Existing studies reveal that the knowledge (both explicit and implicit) endowment heterogeneity of OCC users makes it possible to enrich knowledge resources (Jehn et al., 1997). If such heterogeneity is within a reasonable range, collision, interaction, and fusion between ideas are likely to occur in the process of externalizing knowledge endowment. This helps create an atmosphere for innovation within the community, promote the knowledge exchange and sharing among users, consolidate the collaborative psychology in remix, and offer more possibilities for remix. Finally, there will be more remixes within the community, thus improving the community performance in remix (Rodan and Galunic, 2004; Cao and Yang, 2015; Jin et al., 2021). However, too high knowledge endowment heterogeneity will result in an excessively heterogenous cognition and value judgment among users, a too strong conflicting psychology, and ultimately a reduction in the remix performance within the community. In contrast, too low knowledge endowment heterogeneity will lead to the homogenization of knowledge sources in the community, a decrease of “meta-knowledge” used for remix, and the difficulty to stimulate users’ collaborative psychology. Finally, the community performance in remix will be undermined. Therefore, we propose the following hypotheses:

H7a–H7d: Users’ collaborative psychology plays a mediating role between knowledge endowment heterogeneity (explicit and implicit) and remix (quantity and quality).

H8a–H8d: Users’ conflicting psychology plays a mediating role between knowledge endowment heterogeneity (explicit and implicit) and remix (quantity and quality).

**FIGURE 1:**
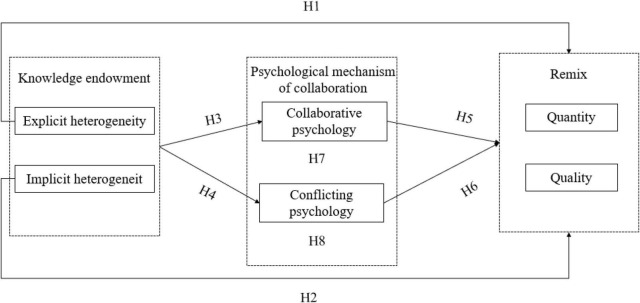
Conceptual model.

On this basis, we propose the following research model as shown in Figure 1.

## Research Design and Methods

### Sampling and Data Collection

The samples were selected from the designs published by Thingiverse users. As the world’s largest OCC, Thingiverse^1^ is a 3D printing community that provides platform components (Hill and Monroy-Hernández, 2013; Flath et al., 2017) to help users in remix. In Thingiverse, you can trace the paths of product remix and evolution. Specifically, citation sources of works are recorded in the remixfrom component, and the remixes component shows the evolution of works remixed by other users. All the platform features can be used for an analysis hereof.

The sample data were acquired through both Python web crawler and manual sorting. Objective data from August 2016 to November 2019 were extracted from the “Explore-Things-Remix” section on the website of Thingiverse. Specific work was carried out in three aspects. Firstly, data of the users and their works in the remix section were collected, and duplicate users were deleted. Secondly, the works information (username, productname, time, posta make, remixes, license, remixfrom, and foundin) and personal information (username and 3Dskilllevel) of sample users were acquired. Thirdly, the users who did not publish any remix (remix value is 0) and whose professional skills were not indicated in the personal information section were removed. Ultimately, 25,032 pieces of related works from 3,004 users were collected for this study.

### Variables

The design of variables was based on previous studies in combination with the platform characteristics of Thingiverse. The specific operational indicators of variables are shown in Table 1.

**TABLE 1:** Measurable indicators and reference sources.

Variable	Measurable indicator	References
Explicit knowledge endowment	Skill level (SL)	Hill and Monroy-Hernández, 2013; Friesike et al., 2019
Implicit knowledge endowment	Quantity of categories of works published by users (FI)	Kyriakou et al., 2017; Friesike et al., 2019
Collaborative psychology	Quantity of works to be remixed (RRG)	Kyriakou et al., 2017
Conflicting psychology	Quantity of works that will not be remixed (NRG)	Friesike et al., 2019
Quantity of remix	Quantity of user works used for remixing (RC)	Stanko, 2016; Feng et al., 2019
Quality of remix	Quantity of user works that are remixed into new works and then printed (RQ)	Friesike et al., 2019
User tenure	Interval (months) between the data collection time and the time of the first product release (TU)	Feng et al., 2019
Original creativity of users	Quantity of original works published by users (UE)	Liu and Sun, 2018

#### Dependent Variables

The quantity of remixes depends on the quantity of works published by Thingiverse users that are used for remixing. The quality of remixes rests on the quantity of user works that are remixed into new works and then printed.

#### Independent Variables

The explicit knowledge endowment heterogeneity is assessed by skill level (SL). As shown on the user information homepage, SL is further divided into three levels, “Novice,” “Intermediate,” and “Advanced,” which are represented by 0, 1, and 2, respectively, in the statistical model. The implicit knowledge endowment heterogeneity is determined by the quantity of categories of works published by users (FI). Thingiverse provides a favorable environment for users to search for innovation inspirations across “domains” and exchange experience and skills with each other. In fact, this community is frequented by “transboundary” works. In other words, a user may publish different categories of works via multiple modules, and different FI represents different levels of implicit knowledge.

#### Intermediary Variables

The collaborative and conflicting psychology of Thingiverse users is reflected by their attitude toward remix. Specifically, users with collaborative psychology may allow others to remix their works, while those with conflicting psychology will refuse others’ remix requests. In the empirical analysis, users’ collaborative psychology can be assessed based on the quantity of published works used by other users for remix, while the conflicting psychology can be analyzed based on the quantity of works that will not be used for remixing. The larger quantity indicates a stronger psychology.

#### Control Variables

User tenure has a noticeable impact on the quantity and quality of remix (Feng et al., 2019). As a control variable, it is measured based on the interval between the data collection time and the time of the first product release (TU). In addition, the relationship of an inverted U-shaped curve exists between the original creativity and remix performance among community users (Kyriakou et al., 2017). As a control variable, original creativity is determined by the quantity of original works published by users (UE).

## Empirical Results and Analysis

### Descriptive Statistical Analysis

Firstly, a descriptive statistical analysis of variables is performed. As shown in Table 2, the maximum, minimum, and mean of the quantity of user works that are remixed into new works and then printed (RQ) are 21, 0, and 0.62, respectively, indicating that the quality of remix by community users is not satisfactory. The maximum and mean of the quantity of user works used for remixing (RC) are 70 and 3.19, respectively, suggesting that the quantity of remix by community users is more satisfactory than the quality. Thus, it can be seen that the management of OCC requires equal attention to the growth rate, quantity, and quality of remix. The mean of SL is 1.23, meaning that most explicit knowledge endowments of community users remain at middle and low levels. The maximum and mean of FI are 8 and 2.78, respectively, indicating the relative richness in categories of works published by community users and the long tail effect in the level of implicit knowledge endowment. Finally, the means of the quantity of works to be remixed (RRG) and the quantity of works that will not be remixed (NRG) are 25.35 and 1.16, respectively, showing that community users’ collaborative psychology is much stronger than the conflicting psychology.

**TABLE 2:** Descriptive statistical analysis.

Indicator	Obs	Mean	SD	Min	Max
SL	3,004	1.23	0.856	0	2
FI	3,004	2.78	1.17	0	8
RRG	3,004	25.35	4.482	0	204
NRG	3,004	1.16	0.971	0	9
RC	3,004	3.19	1.74	1	70
RQ	3,004	0.62	0.74	0	21
TU	3,004	8.17	1.65	0	40
UE	3,004	3.75	1.31	0	21

### Correlation Analysis

As shown in Table 3, the coefficients of correlation between the variables are all < 0.346 and significantly < 0.7, and the main variables are significantly correlated. In addition, variance inflation factors have a mean of 1.14, significantly < 2 and 10, indicating no multicollinearity among the variables.

**TABLE 3:** Correlation analysis.

Indicator	SL	FI	RRG	NRG	RC	RQ
SL	**1.07**					
FI	0.103***	**1.12**				
RRG	0.088***	0.081***	**1.08**			
NRG	0.203***	0.312***	0.095***	**1.25**		
RC	0.189***	0.203***	0.260***	0.346***	−	
RQ	−0.113***	−0.094***	−0.139***	−0.147***	−0.239***	−

***Represent p < 0.001. The diagonal values represent the variance inflation factors of variables.

### Verification of the Structural Equation Model

**FIGURE 2:**
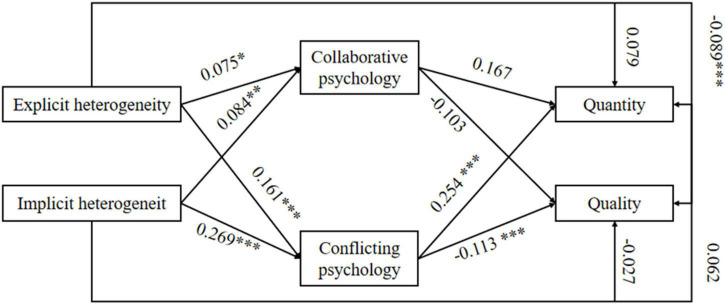
Structural equation model.

The results of fitting analysis on the structural equation model based on Mplus8.1 are as follows: CFI = 0.996, TLI = 0.904, RMSEA = 0.059, and SRMR = 0.011. The degree of fitting is favorable within the critical range. The path coefficient testing results are shown in Table 4, and the structural equation model is shown in Figure 2.

**TABLE 4:** Path analysis and hypothesis testing results of the structural equation model.

Path model	Estimate	S.E.	Est./S.E.	*P*-value
Explicit knowledge endowment→ Collaborative psychology	0.075	0.032	2.350	*
Implicit knowledge endowment→ Collaborative psychology	0.084	0.031	2.677	**
Explicit knowledge endowment→ Conflicting psychology	0.161	0.017	9.362	***
Implicit knowledge endowment→ Conflicting psychology	0.269	0.018	15.265	***
Collaborative psychology→ Quantity	0.167	0.134	1.246	0.213
Collaborative psychology→ Quality	–0.103	0.060	–1.713	0.087
Conflicting psychology→ Quantity	0.254	0.023	11.198	***
Conflicting psychology→ Quality	–0.113	0.029	–3.923	***
Explicit knowledge endowment→ Quantity	0.079	0.047	1.675	0.094
Implicit knowledge endowment→ Quantity	0.062	0.039	1.595	0.111
Explicit knowledge endowment→ Quality	–0.089	0.020	–4.565	***
Implicit knowledge endowment→ Quality	–0.027	0.026	–1.020	0.308

*p < 0.05, **p < 0.01, and ***p < 0.001.

The path coefficient testing results show that the standard errors (S.E.) are all within the theoretical range, without extreme values, thus meeting the requirements. Explicit knowledge endowment heterogeneity (*p* = 0.094 > 0.05) and implicit knowledge endowment heterogeneity (*p* = 0.111 > 0.05) are not significantly associated with the quantity of remix, thus disapproving H1a and H2a. Explicit knowledge endowment heterogeneity is significantly and negatively correlated with the quality of remix (β = −0.089, *p* < 0.001), hence approving H1b, while implicit knowledge endowment heterogeneity is not significantly correlated with the quality of remix (*p* = 0.308 > 0.05), thus disapproving H2b. Moreover, explicit knowledge endowment heterogeneity (β = 0.075, *p* < 0.05) and implicit knowledge endowment heterogeneity (β = 0.084, *p* < 0.01) have a positive impact on users’ collaborative psychology, hence approving H3a and H3b. Explicit knowledge endowment heterogeneity (β = 0.161, *p* < 0.001) and implicit knowledge endowment heterogeneity (β = 0.269, *p* < 0.001) have a positive impact on users’ conflicting psychology, thus approving H4a and H4b. Users’ collaborative psychology is not significantly correlated with the quantity (*p* = 0.213 > 0.05) or quality (*p* = 0.087 > 0.05) of remix, hence disapproving H5a and H5b. Finally, users’ conflicting psychology has a positive impact on the quantity of remix (β = 0.254, *p* < 0.001), hence approving H6a, but is significantly and negatively correlated with the quality of remix (β = −0.113, *p* < 0.001), thus approving H6b.

Users’ collaborative psychology has a negative impact on the quality of remix, which is inconsistent with the hypothesis. This may be attributed to the mechanism of transition from collaborative psychology to conflicting psychology in remix. Firstly, cognitive dissonance plays a mediating role in such transition. The collaborative psychology between users not only diversifies the knowledge elements of products but also reduces the difficulty for users to gain new knowledge. When a user is aware of at least two contradictory cognitive factors during the creation process, he/she will suffer from cognitive dissonance, which will further result in the feeling of tension and even conflict. To alleviate such tension and conflict, the user will try to restore the balance by changing the original cognition, adding new cognition, changing the relative importance of cognition, changing behaviors, etc. Specifically, the user tends to search for works of lower standards, prefer simple mixing and recombination rather than complex creations, and even no longer “pursues perfection” in the search stage. Such transition from collaborative psychology to conflicting psychology has a negative impact on the quality of remix. Secondly, psychological ownership drives such transition. Specifically, when community users generally with collaborative psychology allow other users to remix their knowledge-intensive works for the purpose of innovation, they will desire to monopolize the works, also known as psychological ownership (Feng et al., 2019; Tan et al., 2020). Excessive psychological ownership will entail conflicting psychology and force other users to search for other options of poorer quality for the purpose of remix. As a result, remixing based on simple knowledge works and false prosperity in the quantity of remixes will ultimately reduce the quality of remix and compromise the improvement in quantity and quality of community remixes.

### Verification of the Mediating Effect

We verify the mediating effect using the Bootstrap method (the number of samples is 5,000, with a 95% confidence interval). The significance of the mediating effect depends on whether the confidence interval includes 0, as shown in Table 5. The confidence interval for the indirect effect of users’ conflicting psychology between knowledge endowment heterogeneity (explicit and implicit) and remix (quantity and quality) does not include 0, indicating that the conflicting psychology plays a mediating role between knowledge endowment heterogeneity and remix. However, the results show that the conflicting psychology plays a role of negative mediating role between explicit knowledge endowment heterogeneity and the quality of remix (β = −0.018, *p* < 0.001), and between implicit knowledge endowment heterogeneity and the quality of remix (β = −0.030, *p* < 0.001), which does not approve the basic hypotheses. Therefore, H8a and H8c are approved, while H8b and H8d are disapproved. In addition, the confidence interval for the indirect effect of the collaborative psychology between explicit knowledge endowment heterogeneity and the quantity of remix does not include 0, while the confidence intervals of other paths include 0. Accordingly, H7a, H7c, and H7d are disapproved. Furthermore, the confidence interval of the collaborative psychology between explicit knowledge endowment heterogeneity and the quality of remix does not include 0, meaning that it plays a negative mediating role between explicit knowledge endowment heterogeneity and the quality of remix. This finding is inconsistent with the basic hypotheses, and H7b is disapproved accordingly.

**TABLE 5:** Bootstrap analysis of the mediating effect.

			95% confidence interval			
			No correction		Offset correction	
Path	Estimate	*P*-value	Lower limit	Upper limit	Lower limit	Upper limit
SL→RRG→RC	0.012	0.400	–0.003	0.104	–0.002	0.044
SL→NRG→RC	0.041	***	0.051	0.112	0.030	0.049
FI→RRG→RC	0.014	0.414	–0.002	0.088	–0.002	0.051
FI→NRG→RC	0.068	***	0.064	0.138	0.053	0.078
SL→RRG→RQ	–0.008	0.314	–0.021	–0.001	–0.026	–0.002
SL→NRG→RQ	–0.018	***	–0.021	–0.007	–0.026	–0.010
FI→RRG→RQ	–0.009	0.251	–0.015	–0.001	–0.026	–0.002
FI→NRG→RQ	–0.030	***	–0.025	–0.009	–0.043	–0.017

***Represent p < 0.001.

## Conclusion

### Theoretical Implications

This study looks at user remix in open collaborative communities, builds a model to demonstrate the relationship between user knowledge endowment heterogeneity, collaborative psychology, and remix, and tests the theoretical hypotheses herein by structural equation modeling. The findings are as follows:

Explicit knowledge endowment heterogeneity has no significant impact on the quantity of remixes, but negatively affects the quality. In contrast, implicit knowledge endowment heterogeneity has no significant impact on the quantity and quality of remix. Previous scholars have not reached a consensus on the relationship between knowledge endowment heterogeneity and innovation (Aparna and Hyuntak, 2009). There are three main viewpoints. Firstly, knowledge endowment heterogeneity promotes knowledge sharing, collision, and development among individual users, thereby facilitating innovation (Duan and Yang, 2014). Secondly, knowledge endowment heterogeneity leads to cognitive differences in tasks among users, ultimately reducing the team’s innovation performance (Lovelace et al., 2001). Thirdly, there is an inverted U-shaped relationship between knowledge endowment heterogeneity and innovation, and too high or too low heterogeneity of knowledge endowments hinders the team’s innovation and output (Jetten et al., 1998). However, this empirical study reveals a negative correlation between user knowledge endowment heterogeneity and the quality of remix in OCC, thus expanding the theoretical boundary of the existing literature on the relationship between knowledge endowment heterogeneity and innovation. This suggests the existence of multi-source context effects (Chen, 2011) and multiple explanatory mechanisms (Wang and Li, 2014) between them. Specifically:

(a) There are multiple paths for user knowledge endowment heterogeneity to drive Internet-based innovation. For example, excessive explicit heterogeneity of knowledge endowments among OCC users makes it difficult for other users to understand the connotation and complex relationship between heterogeneous elements and prevents them from absorbing new knowledge effectively. This results in knowledge “overload” and “internal stagnation” of resources (Wu et al., 2022) and undermines their innovation performance. (b) The core and mechanism of remix in the virtual environment, one of the most critical ways of innovation in a prosperous Internet economy, are unique and merit further inquiry. (c) This paper constructs multi-dimensional indicators to measure users’ innovation performance both quantitatively and qualitatively based on the classification of Yan et al. (2019), which may provide a deeper probe into the relationship between knowledge endowment heterogeneity and innovation. (d) This study distinguishes different levels of user knowledge endowment (explicit and implicit, in this study) in the online community (Polanyi, 1958) and enriches the research in knowledge management.

Users’ conflicting psychology plays a mediating role between explicit/implicit knowledge endowment heterogeneity and remix performance (quantity and quality). According to the requisite variety theory, conflict can encourage individuals to generate more viewpoints, ideas, and creativity and improve content innovation (Ashby and Weaver, 2011). Appropriate conflicts help create a favorable atmosphere of group thinking whereby mobilizing individuals to integrate other members’ cognition and viewpoints in their creation, forming a deep fusion of different knowledge and making the content deeper and more affluent (Liu et al., 2018). Users’ collaborative psychology plays a mediating part only between explicit knowledge endowment heterogeneity and remix quality, rather than between explicit knowledge endowment heterogeneity and remix quantity or between implicit knowledge endowment heterogeneity and remix outcome (quantity and quality). Collaboration among users can strengthen knowledge innovation and improve an individual’s innovation performance (Nielsen, 2005). Explicit knowledge endowment heterogeneity provides other users with diverse information and knowledge. The collision of various heterogeneous elements enables users to interact and collaborate, effectively improving the efficiency of knowledge transfer and absorption, breaking through their knowledge limitations, and addressing the isolated islands of knowledge (Wu et al., 2022). In the present study, collaborative and conflicting psychology are deemed two acting forces of the psychological mechanism of collaboration between community users. It focuses on the interrelationship between knowledge endowment heterogeneity, psychological mechanism of collaboration, and remix, explores the mediating role of the psychological mechanism of collaboration between knowledge endowment heterogeneity and remix, and reveals the antecedents, process, and action mechanism that underlying the performance of user remix, hence enriching the perspectives of remix management.

### Practical Implications

The knowledge management of OCC users is expected to focus on the remixing value of user knowledge endowment. Knowledge endowment heterogeneity is a “double-edged sword” (Duan and Yang, 2014), which provides abundant knowledge resources for community remix, on the one hand, and impedes the collaborative interaction between users due to community stratification, on the other hand. Community users can adopt multiple classification standards based on their expertise and search paths. Furthermore, it is necessary to develop and promote a learning mechanism for users with different knowledge endowments and provide a personalized toolkit for the remix by users with varying levels of knowledge and expertise.

The knowledge management of OCC users needs to optimize the psychological mechanism of collaboration further. Stimulating and maintaining users’ collaborative psychology helps boost the interaction and cooperation between users, enhance their sense of community identity and reciprocity, and ultimately improve the remix performance of the community. Controlling the conflicting psychology within a reasonable range may reduce the loss of new users due to too high heterogeneity of knowledge endowments (Qiu and Wang, 2018) and encourage users to reasonably share differentiated knowledge and content, thereby improving the quality and quantity of the overall remix by community users.

The knowledge management of OCC users requires a balance between users’ originality and remix. Maintaining a relatively high originality level of community users helps enrich the “source knowledge” of remixes. However, the excessive novelty will enhance users’ psychological ownership of their works, weaken their collaborating psychology, and consolidate their conflicting psychology. As a result, the prosperous development of remixes will be inhibited.

### Research Limitations and Prospects

The study has limitations in the utilization of objective data. This study is mainly based on the user data recorded on the online platform, thereby having the common limitations in similar studies. In other words, the measurement of each variable is limited by data availability, which will result in such problems as the simplification of variable measurement, insufficiency in control variables, and ex parte consideration of influencing factors. Specifically, users’ implicit knowledge endowments and explicit knowledge endowments can be assessed from multiple dimensions in a more scientific manner. It is impossible to obtain users’ psychological variables only through the assessment of their collaborative and conflicting psychology. In the empirical analysis, only user tenure and original creativity are selected as control variables, while other variables may also play a role. Other user data recorded on Thingiverse include the number of user fans, the number of design teams, the authorization of works, etc. It is necessary to take into account more control variables and make the results more reliable in empirical analysis. In the future, we can obtain psychological variables through questionnaire surveys or experiments and further reveal the mechanism underlying the impact of knowledge endowment heterogeneity on users’ remix behaviors.

Since Thingiverse was upgraded on March 20, 2020, relevant data of remixing sources (remixing from) of community users’ works have been no longer made public, thus significantly affecting the analysis of remix. For this reason, only cross-sectional large sample data from August 2016 to November 2019 were selected for the purpose of the study, thus causing certain limitations to the study. In the future, research conclusions will be more convincing if the data of a larger sample platform with a larger longitudinal interval can be used.

Whereas the empirical analysis data sources are single, the universality of research conclusions needs to be further verified. Future research may be dedicated to a comparative analysis of multiple similar communities, thereby exploring the commonalities and differences in the influencing factors of remix, and further enriching the research conclusions made herein.

## Data Availability Statement

The original contributions presented in this study are included in the article/supplementary material, further inquiries can be directed to the corresponding author/s.

## Author Contributions

JT and XG contributed to conception and design of the study. JT organized the database and wrote the first draft of the manuscript. CQ performed the statistical analysis. XG, JL, and QT wrote sections of the manuscript. All authors contributed to manuscript revision, read, and approved the submitted version.

## Conflict of Interest

The authors declare that the research was conducted in the absence of any commercial or financial relationships that could be construed as a potential conflict of interest.

## Publisher’s Note

All claims expressed in this article are solely those of the authors and do not necessarily represent those of their affiliated organizations, or those of the publisher, the editors and the reviewers. Any product that may be evaluated in this article, or claim that may be made by its manufacturer, is not guaranteed or endorsed by the publisher.

## Footnotes

1
http://www.thingiverse.com
